# 1,4-Bis(3-pyridylmethyl­eneamino­meth­yl)benzene

**DOI:** 10.1107/S1600536809000658

**Published:** 2009-01-10

**Authors:** Ming-Yang He, Chao Li, Huan Xu, Zhao-Jian Hu, Qun Chen

**Affiliations:** a Key Laboratory of Fine Petrochemical Technology, Jiangsu Polytechnic University, Changzhou 213164, People’s Republic of China

## Abstract

The title compound, C_20_H_18_N_4_, is a flexible 3,3′-bipyridyl-type ligand with a long spacer group between the two pyridyl functions. The mol­ecule crystallizes around an inversion center, with one half-mol­ecule in the asymmetric unit and a dihedral angle of 71.85 (8)° between the pyridine ring and the central benzene ring.

## Related literature

For background information on bipyridyl-type Schiff base ligands, see: Cho *et al.* (2006 ▶); Haga *et al.* (1985 ▶); Mahmoudi *et al.* (2007 ▶); Wang *et al.* (2008 ▶). Haga *et al.* (1985 ▶) describe the synthesis of the title compound.
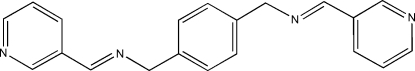


## Experimental

### 

#### Crystal data

C_20_H_18_N_4_
*M* *_r_* = 314.38
Monoclinic, 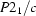

*a* = 6.0990 (11) Å
*b* = 14.589 (3) Å
*c* = 9.9481 (18) Å
β = 107.851 (3)°
*V* = 842.5 (3) Å^3^
*Z* = 2
Mo *K*α radiation
μ = 0.08 mm^−1^
*T* = 291 (2) K
0.24 × 0.22 × 0.20 mm

#### Data collection

Bruker SMART APEX CCD diffractometer
Absorption correction: multi-scan (*SADABS*; Bruker, 2000 ▶) *T*_min_ = 0.98, *T*_max_ = 0.98
6535 measured reflections
1661 independent reflections
1085 reflections with *I* > 2σ(*I*)
*R*_int_ = 0.045

#### Refinement

*R*[*F* ^2^ > 2σ(*F* ^2^)] = 0.048
*wR*(*F* ^2^) = 0.101
*S* = 1.01
1661 reflections
109 parameters
H-atom parameters constrained
Δρ_max_ = 0.13 e Å^−3^
Δρ_min_ = −0.12 e Å^−3^

### 

Data collection: *SMART* (Bruker, 2000 ▶); cell refinement: *SAINT* (Bruker, 2000 ▶); data reduction: *SAINT*; program(s) used to solve structure: *SHELXTL* (Sheldrick, 2008 ▶); program(s) used to refine structure: *SHELXTL*; molecular graphics: *SHELXTL*; software used to prepare material for publication: *SHELXTL*.

## Supplementary Material

Crystal structure: contains datablocks I, global. DOI: 10.1107/S1600536809000658/zl2166sup1.cif

Structure factors: contains datablocks I. DOI: 10.1107/S1600536809000658/zl2166Isup2.hkl

Additional supplementary materials:  crystallographic information; 3D view; checkCIF report

## Figures and Tables

**Fig. 1.**
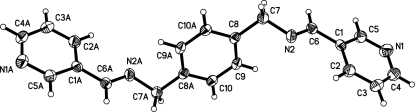
The molecular structure of the title compound (thermal ellipsoids are shown at 30% probability levels).

## supplementary crystallographic information

### Comment

Bipyridyl-type bidentate Schiff base ligands have been utilized intensively to assemble various coordination polymers with interesting topologies and fascinating structural diversities (Cho *et al.*, 2006; Mahmoudi *et al.*, 2007; Wang *et al.*, 2008). We report here the crystal structure of the title compound.

A perspective view of the title compound, including the atomic numbering scheme, is shown in Fig. 1. The title compound crystallizes around a crystallographic center with half a molecule in the asymmetric unit. The bond lengths and angles are within normal ranges. The terminal pyridyl groups are coplanar, and they form a dihedral angle of 71.85 (8)° with the central benzene ring. The molecular structure is stabilized by an intramolecular C9—H9···N2 bond (Table 1), but no classical intermolecular interactions have been found in the crystal packing of the title compound.

### Experimental

The title compound was synthesized and purified according to the method described by by Haga *et al.* (1985), by the condensation reaction of pyridine-3-carboxaldehyde and 1,4-benzenedimethanamine (yield 83%). Colorless block single crystals (m.p. 397–397.2 K) suitable for X-ray analysis were obtained by slow evaporation of a methanol solution at room temperature. Analysis calclated for C_20_H_18_N_4_: C 76.41, H 5.77, N 17.82%; found: C 76.53, H 5.74, N 17.75%. IR (KBr pellet, cm^-1^): 3436 (*b*), 3060 (*m*), 3048 (*m*), 2942 (*m*), 2903 (*m*), 2849 (*m*), 1640 (*s*), 1586 (*s*), 1565 (*s*), 1469 (*s*), 1434 (*s*), 1359 (*m*), 1324 (*m*), 1150 (*w*), 1015 (*m*), 990 (*m*), 848 (*s*), 777 (*s*), 739 (*m*), 617 (*w*), 571 (*m*), 506 (*m*), 403 (*w*).

### Refinement

H atoms were assigned to calculated positions, with C—H = 0.97 (methylene) and 0.93Å (aromatic), and refined using a riding model, with *U*_iso_(H) = 1.2 *U*_eq_(C).

### Crystal data

**Table:**

C_20_H_18_N_4_	*F*(000) = 332
*M**_r_* = 314.38	*D*_x_ = 1.239 Mg m^−^^3^
Monoclinic, *P*2_1_/*c*	Mo *K*α radiation, λ = 0.71073 Å
Hall symbol: -P 2ybc	Cell parameters from 2797 reflections
*a* = 6.0990 (11) Å	θ = 2.6–27.2°
*b* = 14.589 (3) Å	µ = 0.08 mm^−^^1^
*c* = 9.9481 (18) Å	*T* = 291 K
β = 107.851 (3)°	Block, colorless
*V* = 842.5 (3) Å^3^	0.24 × 0.22 × 0.20 mm
*Z* = 2	

### Data collection

**Table:**

Bruker SMART APEX CCD diffractometer	1661 independent reflections
Radiation source: sealed tube	1085 reflections with *I* > 2σ(*I*)
graphite	*R*_int_ = 0.045
φ and ω scans	θ_max_ = 26.0°, θ_min_ = 2.6°
Absorption correction: multi-scan (*SADABS*; Bruker, 2000)	*h* = −7→7
*T*_min_ = 0.98, *T*_max_ = 0.98	*k* = −17→17
6535 measured reflections	*l* = −11→12

### Refinement

**Table:**

Refinement on *F*^2^	Primary atom site location: structure-invariant direct methods
Least-squares matrix: full	Secondary atom site location: difference Fourier map
*R*[*F*^2^ > 2σ(*F*^2^)] = 0.048	Hydrogen site location: inferred from neighbouring sites
*wR*(*F*^2^) = 0.101	H-atom parameters constrained
*S* = 1.01	*w* = 1/[σ^2^(*F*_o_^2^) + (0.04*P*)^2^] where *P* = (*F*_o_^2^ + 2*F*_c_^2^)/3
1661 reflections	(Δ/σ)_max_ < 0.001
109 parameters	Δρ_max_ = 0.13 e Å^−^^3^
0 restraints	Δρ_min_ = −0.12 e Å^−^^3^

### Special details

**Table:**

Geometry. All e.s.d.'s (except the e.s.d. in the dihedral angle between two l.s. planes) are estimated using the full covariance matrix. The cell e.s.d.'s are taken into account individually in the estimation of e.s.d.'s in distances, angles and torsion angles; correlations between e.s.d.'s in cell parameters are only used when they are defined by crystal symmetry. An approximate (isotropic) treatment of cell e.s.d.'s is used for estimating e.s.d.'s involving l.s. planes.
Refinement. Refinement of *F*^2^ against ALL reflections. The weighted *R*-factor *wR* and goodness of fit *S* are based on *F*^2^, conventional *R*-factors *R* are based on *F*, with *F* set to zero for negative *F*^2^. The threshold expression of *F*^2^ > σ(*F*^2^) is used only for calculating *R*-factors(gt) *etc*. and is not relevant to the choice of reflections for refinement. *R*-factors based on *F*^2^ are statistically about twice as large as those based on *F*, and *R*- factors based on ALL data will be even larger.

### Fractional atomic coordinates and isotropic or equivalent isotropic displacement parameters (Å^2^)

**Table:**

	*x*	*y*	*z*	*U*_iso_*/*U*_eq_	
C1	0.0338 (3)	0.59733 (9)	0.90988 (16)	0.0402 (4)	
C2	0.2284 (3)	0.63315 (11)	1.00966 (18)	0.0512 (4)	
H2	0.3749	0.6142	1.0117	0.061*	
C3	0.2005 (3)	0.69698 (11)	1.1050 (2)	0.0592 (5)	
H3	0.3278	0.7216	1.1728	0.071*	
C4	−0.0183 (4)	0.72359 (11)	1.0983 (2)	0.0627 (5)	
H4	−0.0346	0.7673	1.1625	0.075*	
C5	−0.1780 (3)	0.62821 (11)	0.91503 (19)	0.0522 (4)	
H5	−0.3091	0.6036	0.8506	0.063*	
C6	0.0482 (3)	0.53005 (10)	0.80278 (16)	0.0418 (4)	
H6	−0.0874	0.5064	0.7416	0.050*	
C7	0.2348 (3)	0.43652 (11)	0.68220 (17)	0.0475 (4)	
H7A	0.0771	0.4255	0.6247	0.057*	
H7B	0.2981	0.3791	0.7264	0.057*	
C8	0.3738 (3)	0.46922 (10)	0.58876 (15)	0.0390 (4)	
C9	0.3934 (3)	0.56235 (10)	0.56217 (16)	0.0430 (4)	
H9	0.3223	0.6050	0.6044	0.052*	
C10	0.5155 (3)	0.59265 (10)	0.47492 (17)	0.0424 (4)	
H10	0.5239	0.6551	0.4585	0.051*	
N1	−0.2079 (3)	0.69096 (10)	1.00596 (19)	0.0667 (5)	
N2	0.2369 (2)	0.50359 (9)	0.79117 (15)	0.0489 (4)	

### Atomic displacement parameters (Å^2^)

**Table:**

	*U*^11^	*U*^22^	*U*^33^	*U*^12^	*U*^13^	*U*^23^
C1	0.0437 (9)	0.0402 (7)	0.0394 (9)	−0.0009 (6)	0.0165 (7)	0.0070 (6)
C2	0.0458 (10)	0.0571 (10)	0.0500 (10)	0.0002 (8)	0.0134 (8)	−0.0032 (8)
C3	0.0643 (12)	0.0516 (10)	0.0579 (12)	−0.0079 (8)	0.0134 (9)	−0.0118 (8)
C4	0.0786 (14)	0.0431 (9)	0.0738 (13)	−0.0012 (9)	0.0345 (11)	−0.0131 (9)
C5	0.0470 (9)	0.0501 (9)	0.0600 (11)	0.0011 (8)	0.0169 (8)	−0.0034 (8)
C6	0.0425 (9)	0.0458 (8)	0.0374 (9)	−0.0011 (6)	0.0127 (7)	0.0029 (6)
C7	0.0467 (9)	0.0508 (8)	0.0462 (9)	0.0005 (7)	0.0161 (7)	−0.0041 (7)
C8	0.0379 (8)	0.0423 (7)	0.0333 (9)	0.0013 (6)	0.0055 (6)	−0.0063 (6)
C9	0.0464 (9)	0.0423 (7)	0.0414 (9)	0.0065 (7)	0.0149 (7)	−0.0081 (7)
C10	0.0477 (9)	0.0346 (7)	0.0442 (9)	0.0017 (6)	0.0129 (7)	−0.0025 (6)
N1	0.0609 (10)	0.0550 (9)	0.0887 (13)	0.0041 (8)	0.0297 (9)	−0.0173 (8)
N2	0.0478 (8)	0.0624 (9)	0.0393 (8)	0.0002 (6)	0.0175 (6)	−0.0074 (6)

### Geometric parameters (Å, °)

**Table:**

C1—C5	1.384 (2)	C6—H6	0.9300
C1—C2	1.395 (2)	C7—N2	1.458 (2)
C1—C6	1.471 (2)	C7—C8	1.514 (2)
C2—C3	1.376 (2)	C7—H7A	0.9700
C2—H2	0.9300	C7—H7B	0.9700
C3—C4	1.372 (3)	C8—C10^i^	1.391 (2)
C3—H3	0.9300	C8—C9	1.396 (2)
C4—N1	1.325 (3)	C9—C10	1.378 (2)
C4—H4	0.9300	C9—H9	0.9300
C5—N1	1.338 (2)	C10—C8^i^	1.391 (2)
C5—H5	0.9300	C10—H10	0.9300
C6—N2	1.253 (2)		
			
C5—C1—C2	116.88 (15)	N2—C7—C8	111.49 (13)
C5—C1—C6	120.51 (15)	N2—C7—H7A	109.3
C2—C1—C6	122.61 (14)	C8—C7—H7A	109.3
C3—C2—C1	119.09 (16)	N2—C7—H7B	109.3
C3—C2—H2	120.5	C8—C7—H7B	109.3
C1—C2—H2	120.5	H7A—C7—H7B	108.0
C4—C3—C2	118.78 (18)	C10^i^—C8—C9	117.60 (13)
C4—C3—H3	120.6	C10^i^—C8—C7	121.10 (13)
C2—C3—H3	120.6	C9—C8—C7	121.30 (12)
N1—C4—C3	124.15 (17)	C10—C9—C8	121.72 (13)
N1—C4—H4	117.9	C10—C9—H9	119.1
C3—C4—H4	117.9	C8—C9—H9	119.1
N1—C5—C1	124.74 (18)	C9—C10—C8^i^	120.68 (13)
N1—C5—H5	117.6	C9—C10—H10	119.7
C1—C5—H5	117.6	C8^i^—C10—H10	119.7
N2—C6—C1	122.21 (15)	C4—N1—C5	116.34 (16)
N2—C6—H6	118.9	C6—N2—C7	118.48 (14)
C1—C6—H6	118.9		

Symmetry codes: (i) −*x*+1, −*y*+1, −*z*+1.

### Hydrogen-bond geometry (Å, °)

**Table:**

*D*—H···*A*	*D*—H	H···*A*	*D*···*A*	*D*—H···*A*
C9—H9···N2	0.93	2.55	2.858 (2)	100

## References

[bb1] Bruker (2000). *SADABS* and *SAINT* . Bruker AXS Inc., Madison, Wisconsin, USA.

[bb2] Cho, B. Y., Min, D. W. & Lee, S. W. (2006). *Cryst. Growth Des.* **6**, 342–347.

[bb3] Haga, M. & Koizumi, K. (1985). *Inorg. Chim. Acta*, **104**, 47–50.

[bb4] Mahmoudi, G., Morsali, A., Hunter, A. D. & Zeller, M. (2007). *CrystEngComm*, **9**, 704–714.

[bb5] Sheldrick, G. M. (2008). *Acta Cryst.* A**64**, 112–122. 10.1107/S0108767307043930 18156677

[bb6] Wang, Q., Yang, R., Zhuang, C. F., Zhang, J. Y., Kang, B. S. & Su, C. Y. (2008). *Eur. J. Inorg. Chem.* **10**, 1702–1711.

